# Performance of Large Language Models in Diagnosing Rare Hematologic Diseases and the Impact of Their Diagnostic Outputs on Physicians: Combined Retrospective and Prospective Study

**DOI:** 10.2196/77334

**Published:** 2025-10-09

**Authors:** Hongbin Yu, Tian Chen, Xin Zhang, Yunfan Yang, Qinyu Liu, Chenlu Yang, Kai Shen, He Li, Wenjiao Tang, Xushu Zhong, Xiao Shuai, Xinmei Yu, Yi Liao, Chiyi Wang, Huanling Zhu, Yu Wu

**Affiliations:** 1 Department of Hematology, West China Hospital of Sichuan University, Guoxue Alley 37th, Chengdu, 610041, China, 86 102885422370, 86 102885423692

**Keywords:** rare hematologic disease, large language model, LLM, hematology, ChatGPT, artificial intelligence, AI

## Abstract

**Background:**

Rare hematologic diseases are frequently underdiagnosed or misdiagnosed due to their clinical complexity. Whether new-generation large language models (LLMs), particularly those using chain-of-thought reasoning, can improve diagnostic accuracy remains unclear.

**Objective:**

This study aimed to evaluate the diagnostic performance of new-generation commercial LLMs in rare hematologic diseases and to determine whether the LLM output enhances physicians’ diagnostic accuracy.

**Methods:**

We conducted a 2-phase study. In the retrospective phase, we evaluated 7 mainstream LLMs on 158 nonpublic real-world admission records covering 9 rare hematologic diseases, assessed diagnostic performance using top-10 accuracy and mean reciprocal rank (MRR), and evaluated ranking stability via Jaccard similarity and entropy. Spearman rank correlation was used to examine the association between physicians’ diagnoses and LLM-generated outputs. In the prospective phase, 28 physicians with varying levels of experience diagnosed 5 cases each, gaining access to LLM-generated diagnoses across 3 sequential steps to assess whether LLMs can improve diagnostic accuracy.

**Results:**

In the retrospective phase, ChatGPT-o1-preview demonstrated the highest top-10 accuracy (70.3%) and MRR (0.577), and DeepSeek-R1 ranked second. Diagnostic performance was low for amyloid light-chain (AL) amyloidosis; Castleman disease; Erdheim-Chester disease; and polyneuropathy, organomegaly, endocrinopathy, monoclonal gammopathy, and skin changes (POEMS) syndrome. Interestingly, higher accuracy often correlated with lower ranking stability across most LLMs. The physician performance showed a strong correlation with both top-10 accuracy (ρ=0.565) and MRR (ρ=0.650). In the prospective phase, LLMs significantly improved the diagnostic accuracy of less-experienced physicians; no significant benefit was observed for specialists. However, when LLMs generated biased responses, physician performance often failed to improve or even declined.

**Conclusions:**

Without fine-tuning, new-generation commercial LLMs, particularly those with chain-of-thought reasoning, can identify diagnoses of rare hematologic diseases with high accuracy and significantly enhance the diagnostic performance of less-experienced physicians. Nevertheless, biased LLM outputs may mislead clinicians, highlighting the need for critical appraisal and cautious clinical integration with appropriate safeguard systems.

## Introduction

### Background

Rare diseases, or orphan diseases, are defined as conditions with extremely low prevalence. Their low incidence and diverse clinical manifestations pose significant diagnostic challenges. Many rare diseases lack distinct clinical features, leading to frequent misdiagnoses or missed diagnoses. Most clinicians may only encounter a few such cases throughout their careers, resulting in limited diagnostic experience [1,2]. The same difficulties apply to rare hematologic diseases, especially those that occur during adulthood; such adult-onset diseases have prolonged diagnostic periods, tend to involve multiple organ systems, have an insidious onset, and are often diagnosed only at an advanced stage [3-7].

With the rapid development of artificial intelligence (AI)—particularly transformer-based large language models (LLMs)—researchers have begun investigating their applications in clinical settings including medical diagnosis [8-10]. Current findings show that LLMs perform similarly to physicians in diagnosing common diseases. Given that rare diseases are encountered less frequently by general physicians compared to common conditions, LLMs may offer a unique advantage in their diagnosis. Interestingly, it was recently reported in the news how a patient’s family used ChatGPT, along with physician-provided clinical data, to diagnose a long-misdiagnosed, rare congenital neurologic disease [11]. This example suggests that publicly available LLMs, even those not specifically optimized for clinical diagnosis, already demonstrate practical medical utility. Other studies conducted by professionals also indicated that widely accessible LLMs can assist in diagnosing rare diseases [12-16], suggesting that LLMs may offer greater clinical benefits in the diagnosis of rare diseases compared to common conditions. As publicly available LLMs evolve rapidly, chain-of-thought (CoT) methods have been introduced in commercial releases, enabling models to generate step-by-step intermediate reasoning rather than immediate answers. This approach helps clarify complex problems and improves both reasoning accuracy and explainability [17]. Since early 2025, models such as DeepSeek-R1 and ChatGPT-o1 have made their CoT processes publicly accessible, enabling users to trace and evaluate diagnostic logic [18,19]. The disclosure of CoT reasoning offers distinct advantages in medical diagnosis. By revealing intermediate steps, CoT not only yields a final diagnosis but also provides a transparent rationale. This enables health care professionals to trace the model’s decision-making process, supporting validation and clinical review. By enhancing diagnostic robustness through self-consistency, CoT mitigates the black box effect, reduces the risk of misdiagnosis, and improves the safety of clinical applications [20,21].

Despite these advancements, several questions remain about using LLMs to diagnose rare diseases. Most existing studies are retrospective and involve limited case numbers, and none have evaluated LLMs in rare hematologic diseases. Current evaluations largely rely on public datasets rather than unreleased real-world clinical records, raising concerns about potential training data leakage and limiting generalizability due to preprocessing in standardized corpora. In addition, much of the literature focuses on older models, such as ChatGPT-4, while few assessments have examined newer-generation LLMs, particularly those incorporating CoT reasoning, which may improve diagnostic accuracy and transparency. Furthermore, even when retrospective analyses suggest promising accuracy, integrating LLMs into routine clinical workflows remains challenging. How LLM-derived diagnoses influence physician decision-making, especially with the introduction of CoT reasoning, is still poorly understood. To date, no prospective study has evaluated whether LLMs can improve physician diagnostic performance in rare diseases, let alone in rare hematologic diseases [22-26].

### Goal of This Study

To address these gaps, we conducted a retrospective study using deidentified real-world admission records from our center. These records were input into non-fine-tuned, newer-generation, publicly available, commercially deployable LLMs, including those with CoT capabilities, to generate their top 10 likely diagnoses, which were then assessed for diagnostic performance. We also prospectively presented the top 10 likely diagnoses generated by the highest-performing LLM, followed step-by-step by its reasoning and analysis results, to physicians with varying levels of experience in order to assess whether this information could enhance their ability to diagnose rare hematologic diseases. This prospective component simulated real-world physician-LLM interactions to examine practicality and safety, providing a potential pathway for clinical deployment and representing a critical step in translating LLMs from research tools to clinical applications.

## Methods

### Ethical Considerations

This study was conducted in accordance with the Declaration of Helsinki, approved by the institutional review board of West China Hospital, Sichuan University (2024‐1754), registered in the Chinese Clinical Trial Registry (ChiCTR2400089959), and reported following the Standards for Reporting Diagnostic Accuracy guidelines [27]. Informed consent was waived by the institutional review board, which also approved the secondary analysis of patient data without additional consent. To ensure privacy and confidentiality, all patient records were deidentified, and no identifiable personal information was included in the study. During the prospective phase, each participating physician received approximately US $14 (¥100 RMB) as monetary compensation.

### Retrospective Phase

#### Input Data Collection

The detailed information regarding the methods used is provided in Multimedia Appendix 1. We selected all 25 rare hematologic diseases from the first and second People’s Republic of China national lists of rare diseases [28,29], all of which also meet the rare disease criteria in the United States and Europe [30,31]. We then searched our hospital information system for inpatients with a primary diagnosis of any of these diseases. Following China’s previous rare disease information management regulations, up to 20 patient records were included per disease [32], selected in reverse chronological order. Only the first hospitalization related to the rare disease was included, regardless of whether the diagnosis was confirmed at discharge. Cases lacking definitive diagnostic evidence in the final discharge summary (eg, missing confirmatory tests) or involving day-case procedures were excluded. Diseases with fewer than 8 total cases were omitted. All final diagnoses were confirmed according to the standard diagnostic criteria [4,33-40].

#### Data Preparation and LLM Evaluation

From each patient’s record, we removed any definitive diagnostic statements (admitting, imaging, and pathological diagnoses) and personal identifiers but retained descriptive findings from imaging or pathology reports. The processed records were compiled into a standardized table in their original Chinese language, without translation, for subsequent LLM analysis. We then evaluated 7 publicly available LLMs via their official application programming interfaces: Claude 3.5 Sonnet (claude-3‐5-sonnet-20241022; Anthropic PBC), DeepSeek-R1 (deepseek-reasoner; DeepSeek), Doubao (Doubao-1.5-Pro-256k; ByteDance), Gemini Experimental 1206 (gemini-exp-1206; Google LLC), ChatGPT-4o (gpt-4o-2024-11-20; OpenAI), ChatGPT-o1-preview (o1-preview-2024-09-12; OpenAI), and Qwen (Qwen-Max-2025-01-25). Each model, using default settings, was asked to provide the top 10 likely diagnoses for each case in 5 repeated responses. All models were prompted in Chinese as follows:

*Please assume you are an experienced professional physician. Here is an admission medical record. Based on its content, provide the 10 most likely main diagnoses, ranked from most to least probable.*

We then used the gpt-4o-2024-11-20 model to standardize and merge identical or similar diagnoses for each patient record. The merged diagnoses were subsequently reviewed by 2 physicians (HY and TC) to ensure no incorrect merges and to assess diagnostic correctness. If a correct diagnosis was identified among the list, we standardized its name for subsequent statistical analyses. The *correct* diagnosis had to capture the essential features of the disease. All judgments were independently cross-validated by 2 physicians (HY and TC).

#### Outcome Measures

For each model, we calculated the top-10 accuracy (the proportion of cases in which the correct diagnosis was present among the model’s top 10 suggestions) and the mean reciprocal rank (MRR) of the correct diagnosis. To explore potential sources of variation in LLM performance across diseases, we also measured the frequency of diagnostic keywords per 1000 Chinese characters to determine diagnostic keyword density. We assessed the stability of model outputs across the 5 runs for a given case using Jaccard similarity coefficients and entropy values (higher entropy indicating more variability). We also compared model performance with physician performance. Each case’s initial physician diagnosis from the admission record was rated on a 5-point accuracy scale: 5 points for a completely correct diagnosis, 4 points if the diagnosis was in the correct direction, 3 points if there was no diagnostic error and the description primarily outlined symptoms objectively, 2 points if the diagnosis demonstrated an incorrect tendency, and 1 point for a completely incorrect diagnosis (Table S1 in Multimedia Appendix 1). We calculated Spearman correlation coefficients to examine the association between the physicians’ scores and the LLMs’ diagnostic accuracies.

### Prospective Evaluation

Then, we conducted a prospective study to test whether LLM-provided diagnoses and reasoning could aid physician diagnosis. In total, 5 cases, each representing a different rare hematologic disease from the retrospective cohort, were randomly assigned to each participant in this phase. Using the same prompts, we accessed ChatGPT-o1 via its web interface and required additional analysis aligned with that used in the retrospective application programming interface. Each output included a diagnosis list, explanatory analysis, and detailed CoT reasoning (translated into Chinese when necessary). We determined the sample size using power analysis with G*Power (version 3.1.9.7; Heinrich-Heine-Universität Düsseldorf). Under the settings of *F* tests, ANOVA repeated measures, within-between interaction, and a priori analysis, we specified an effect size (*f*) of 0.25, an α error probability of 0.05, a power of 0.8, a correlation among repeated measures of 0.7, and a nonsphericity correction ε of 1. Assuming one question per participant in a 4-group, 3-timepoint design, the calculated total sample size was 28, which we subsequently enrolled. Physicians from our institution (7 per experience level: postresidency physicians, nonhematology attendings, hematology attendings, and consultant hematologists) were recruited to participate. Each physician independently evaluated all 5 cases via an online system. For each case, the physician first reviewed the admission record and provided an initial diagnosis without any AI assistance. They then saw the LLM’s top 10 suggested diagnoses and gave a second diagnosis. Next, the LLM’s step-by-step reasoning and analysis were shown, after which the physician provided a third (final) diagnosis. Finally, the true diagnosis was revealed, and the physician rated the usefulness of the LLM’s information on a 5-point scale mentioned earlier (Tables S2 and S3 in Multimedia Appendix 1). Once initiated, participants were not allowed to revisit previous pages.

### Statistical Analysis

In the retrospective study, one-way ANOVA was used for group comparisons. The Spearman rank correlation coefficient (*ρ*) was used to assess the association between physician scores and both the top-10 diagnostic list and MRR.

In this prospective study, we compared physicians’ diagnostic performance before and after LLM assistance using paired nonparametric tests. Differences among the 4 experience groups were evaluated with appropriate nonparametric analyses. To assess LLM impact, cases where LLM failed to produce the correct diagnosis were defined as biased. Improvement was defined as a correct diagnosis appearing in the second or third response; otherwise, no change or a decrease was defined as unchanged or declined. Subjective ratings of 4‐5 were positive, 3 was neutral, and 1‐2 were negative. Firth logistic regression was used to address data separation. For subjective ratings violating the Brant test, we applied nominal multinomial regression. A 2-sided *P* value <.05 was considered statistically significant. Analyses were conducted using Python (version 3.13.1; Python Software Foundation); R (version 4.4.2; R Foundation for Statistical Computing); and GraphPad Prism (version 10.4; Dotmatics).

## Results

### Retrospective Phase

#### Enrollment

**Figure 1.**
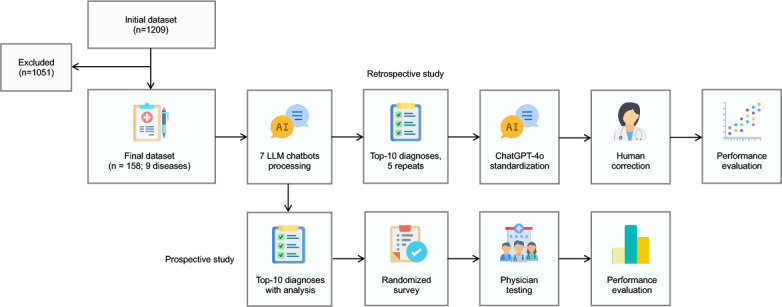
Study design, including a retrospective analysis of large language model (LLM) diagnostic performance and a prospective evaluation of the impact of LLM-generated information on physician performance.

We conducted this study following the design outlined in Figure 1. In compliance with previous regulations on rare disease information management of China and its associated case number limitations [32], we included admission records of 158 patients diagnosed with 9 rare hematologic diseases from our center: amyloid light-chain (AL) amyloidosis; Castleman disease; Erdheim-Chester disease (ECD); polyneuropathy, organomegaly, endocrinopathy, monoclonal gammopathy, and skin changes (POEMS) syndrome; Waldenström macroglobulinemia; acquired hemophilia; Langerhans cell histiocytosis (LCH); cutaneous T-cell lymphoma; and thrombotic thrombocytopenic purpura. After removal of identifying and diagnostic information, each case’s admission record was processed by 7 LLMs using default parameters. Among these models, Claude 3.5 Sonnet, DeepSeek-R1, and ChatGPT-o1-preview officially feature CoT capabilities [41]. We prompted each model to generate the 10 most likely primary diagnoses in descending order of likelihood.

#### Top-10 Accuracy

**Figure 2.**
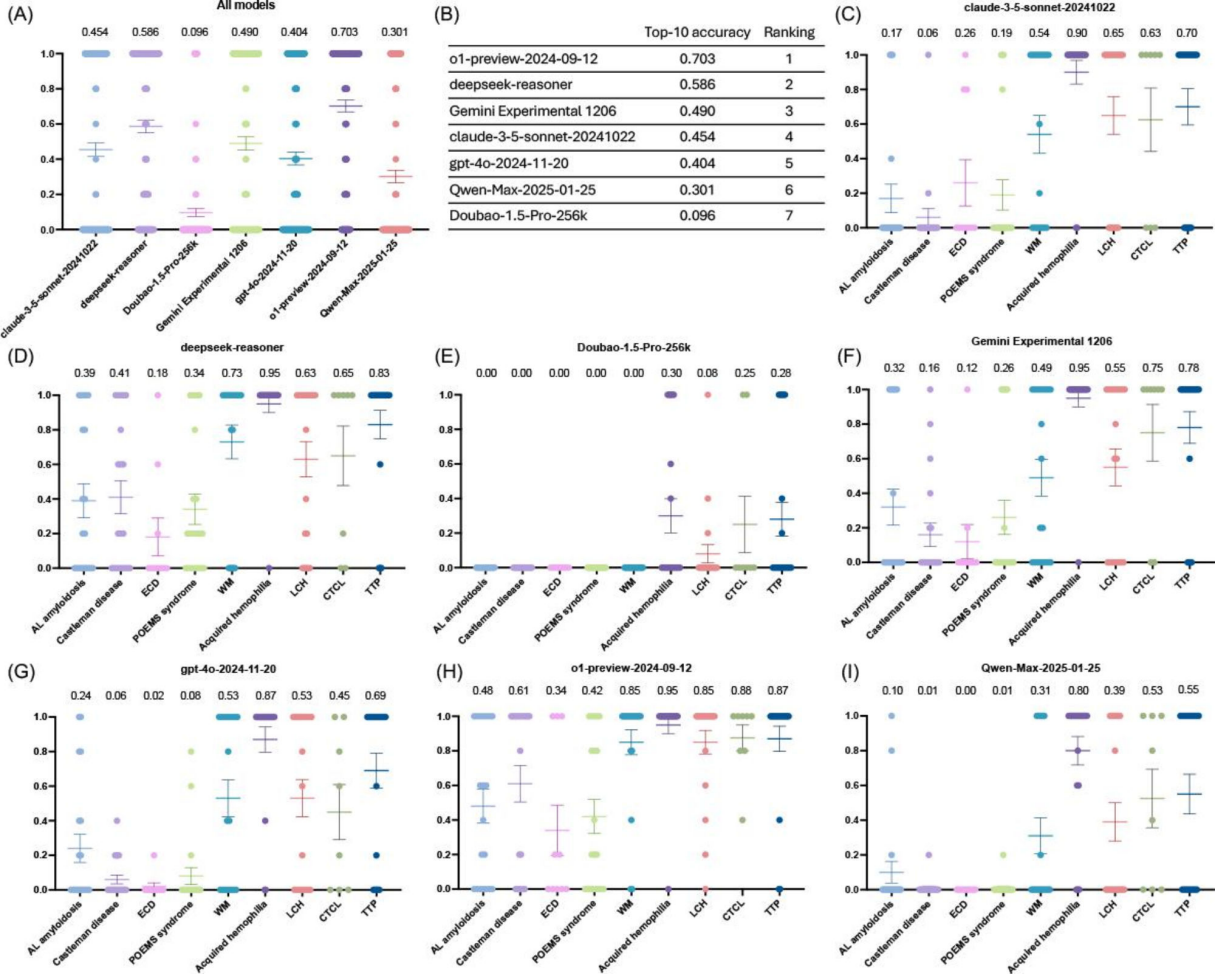
Top-10 accuracy of different large language models (LLMs) in diagnosing rare hematologic diseases. (A and B) Overall top-10 accuracy of selected LLMs. (C–I) Top-10 accuracy of LLM models for different rare hematologic diseases: (C) Claude 3.5 Sonnet, (D) DeepSeek-R1, (E) Doubao-1.5-Pro-256k, (F) Gemini Experimental 1206, (G) ChatGPT-4o, (H) ChatGPT-o1-preview, (I) Qwen-Max-2025-01-25. AL amyloidosis: amyloid light-chain amyloidosis; CTCL: cutaneous T-cell lymphoma; ECD: Erdheim-Chester disease; LCH: Langerhans cell histiocytosis; POEMS: polyneuropathy, organomegaly, endocrinopathy, monoclonal gammopathy, and skin changes; TTP: thrombotic thrombocytopenic purpura; WM: Waldenström macroglobulinemia.

The top-10 accuracy (score range 0‐1) results indicated that ChatGPT-o1-preview performed best (0.703), successfully including the correct diagnosis in 70.3% of cases when generating 10 differential diagnoses. It was followed by DeepSeek-R1, Gemini Experimental 1206, Claude 3.5 Sonnet, ChatGPT-4o, Qwen-Max-2025-01-25, and Doubao-1.5-Pro-256k. Notably, all models scored low for AL amyloidosis, Castleman disease, ECD, and POEMS syndrome, while they performed better for Waldenström macroglobulinemia, acquired hemophilia, LCH, cutaneous T-cell lymphoma, and thrombotic thrombocytopenic purpura—especially acquired hemophilia (Figure 2; Table S4 in Multimedia Appendix 1).

#### Mean Reciprocal Rank

**Figure 3.**
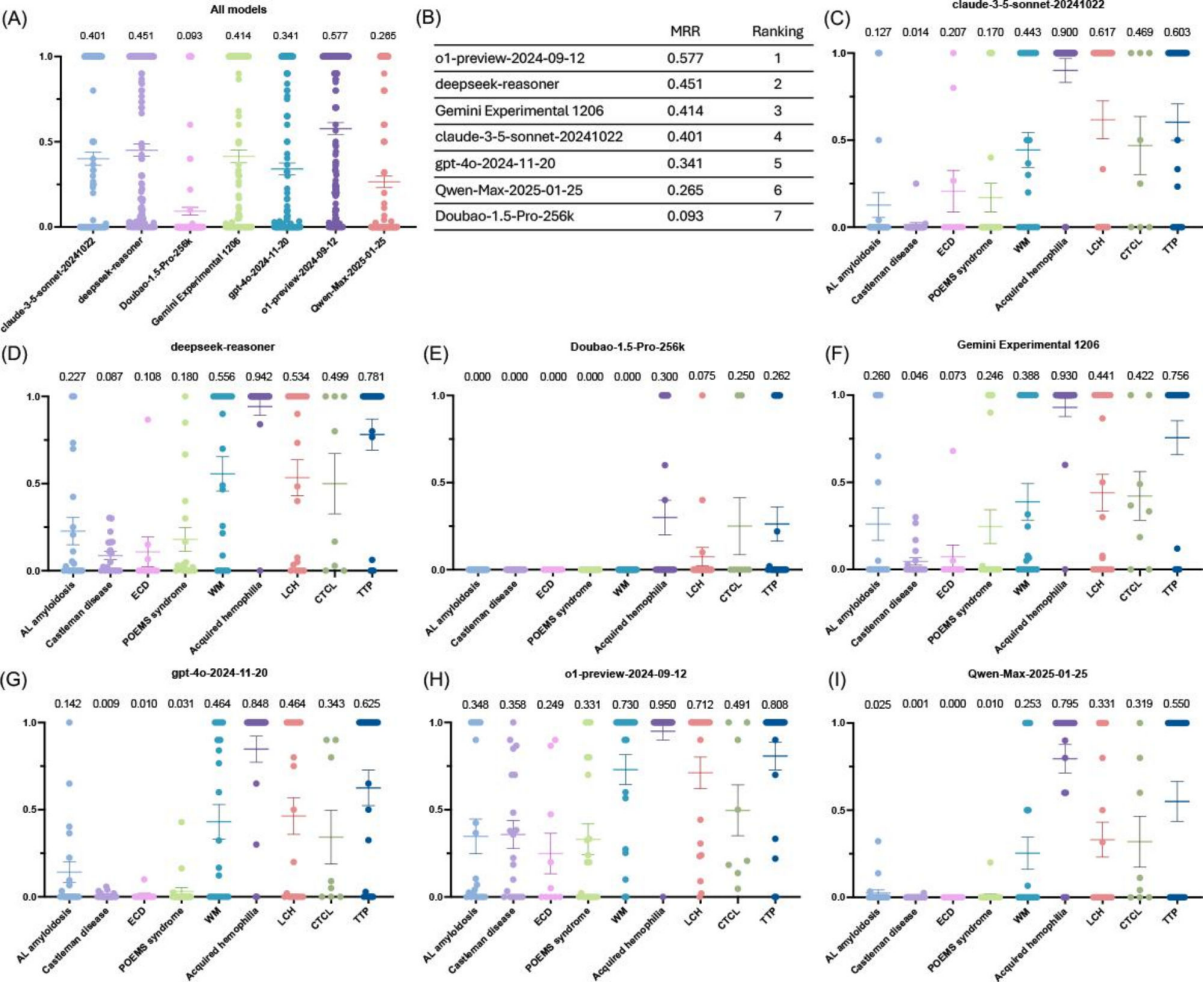
Mean reciprocal rank (MRR) of different large language models (LLMs) in diagnosing rare hematologic diseases. (A and B) Overall MRR of selected LLMs. (C–I) MRR of LLM models for different rare hematologic diseases: (C) Claude 3.5 Sonnet, (D) DeepSeek-R1, (E) Doubao-1.5-Pro-256k, (F) Gemini Experimental 1206, (G) ChatGPT-4o, (H) ChatGPT-o1-preview, (I) Qwen-Max-2025-01-25. AL amyloidosis: amyloid light-chain amyloidosis; CTCL: cutaneous T-cell lymphoma; ECD: Erdheim-Chester disease; LCH: Langerhans cell histiocytosis; POEMS: polyneuropathy, organomegaly, endocrinopathy, monoclonal gammopathy, and skin changes; TTP: thrombotic thrombocytopenic purpura; WM: Waldenström macroglobulinemia.

The MRR (score range 0‐1) results followed a similar pattern of top-10 accuracy. ChatGPT-o1-preview again achieved the highest MRR (0.577), indicating superior performance by placing the correct diagnosis closer to the top among the 10 generated differential diagnoses. The next best performers were DeepSeek-R1, Gemini Experimental 1206, Claude 3.5 Sonnet, ChatGPT-4o, Qwen-Max-2025-01-25, and Doubao-1.5-Pro-256k. All models exhibited lower MRRs compared to top-10 accuracy, suggesting that correct diagnoses were not consistently ranked first. This aligns with typical clinical reasoning, where common diseases are considered before rare ones. Consistent with top-10 accuracies, all models performed relatively poorly on AL amyloidosis, Castleman disease, ECD, and POEMS syndrome (Figure 3; Table S5 in Multimedia Appendix 1). To better elucidate the reasons for the lower diagnostic performance of LLMs on certain diseases, we searched the PubMed database using standardized disease names to quantify the number of related publications (search conducted on September 10, 2025). The results indicated that 4 of the more difficult-to-diagnose diseases had substantially fewer publications compared with better-performing diseases, with the exception of acquired hemophilia (Figure S1 in Multimedia Appendix 1). We then analyzed diagnostic keyword density in admission records to further examine this discrepancy. The results demonstrated that these 4 difficult-to-diagnose diseases exhibited significantly lower keyword density than other diseases (Figure S2 in Multimedia Appendix 1), a pattern consistent with the diagnostic performance of LLMs. These results indicate that corpus richness and diagnostic keyword density in clinical records significantly influence the diagnostic capability of LLMs for rare diseases.

#### Stability

**Figure 4.**
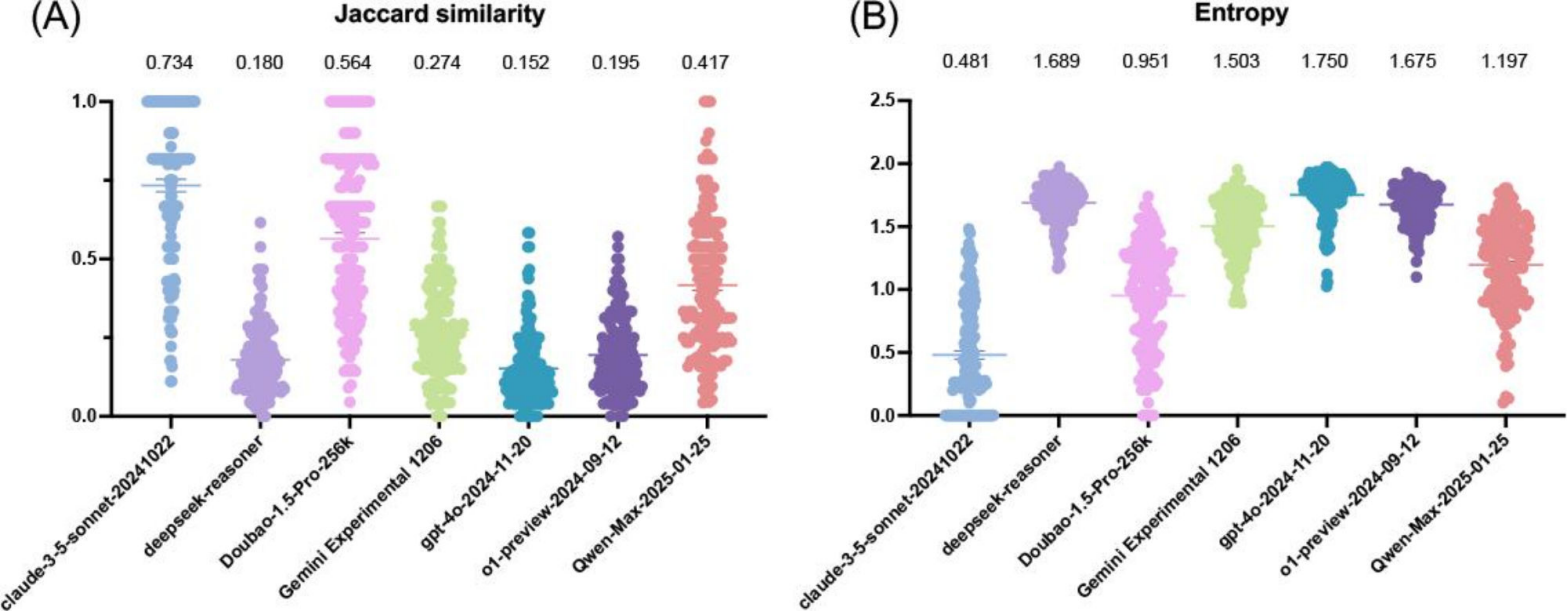
Stability of large language model diagnostic outputs for rare hematologic diseases. Stability was evaluated using (A) Jaccard similarity and (B) entropy across models. Higher accuracy generally coincided with lower stability, except for Claude 3.5 Sonnet.

Regarding stability, ChatGPT-4o demonstrated the broadest range of returned diagnoses and the lowest stability, with a Jaccard similarity (score range 0‐1) of 0.152, where lower values indicate less consistency across outputs. DeepSeek-R1 ranked next, followed by ChatGPT-o1-preview, Gemini Experimental 1206, Qwen-Max-2025-01-25, Doubao-1.5-Pro-256k, and Claude 3.5 Sonnet. Entropy values (ranging from 0 to 2.3026) also identified ChatGPT-4o as least stable (1.750), as higher values imply more random rankings. The next models were DeepSeek-R1, ChatGPT-o1-preview, Gemini Experimental 1206 (1.507), Qwen-Max-2025-01-25, Doubao-1.5-Pro-256k, and Claude 3.5 Sonnet, closely mirroring the Jaccard similarity results. Taken together, these findings suggest that higher accuracy often corresponds to lower stability across most LLMs, except for Claude 3.5 Sonnet, which maintained both relatively high accuracy and stable outputs (Figure 4; Tables S6 and S7 in Multimedia Appendix 1).

#### Outcome Measures

**Figure 5.**
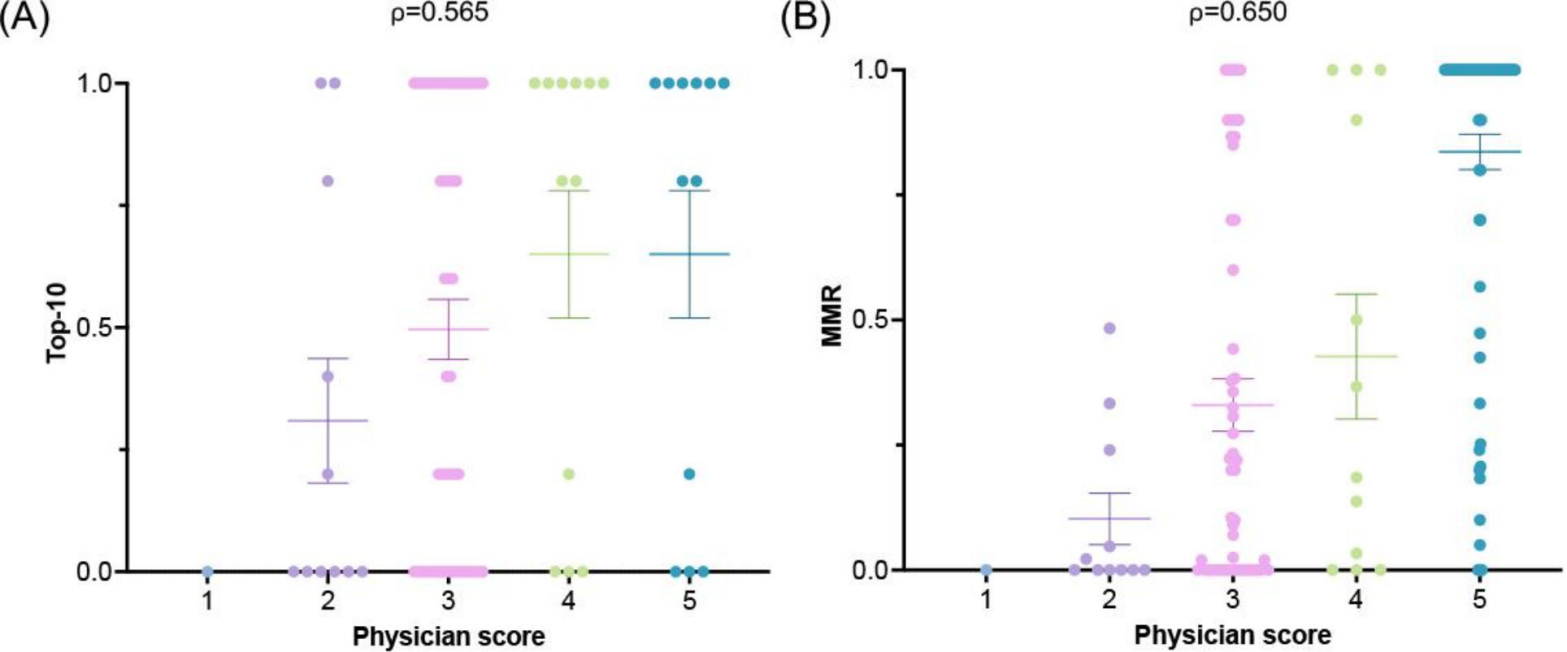
Spearman rank correlation between physician diagnostic performance and large language model outputs. (A) Correlation between physician scores and top-10 accuracy. (B) Correlation between physician scores and mean reciprocal rank (MRR). Physician performance showed a strong correlation with both metrics.

We next explored correlations between human physician scores and LLM diagnostic outputs. A 5-point rating system was used to evaluate physician diagnoses, where a score of 3 indicated a neutral result with no significant errors (Table S1 in Multimedia Appendix 1). Physicians achieved 92.4% (146/158) accuracy for scores ≥3 and 59.5% (94/158) for scores of 4 and 5. For LLM outputs, we examined Spearman rank correlation between physician performance and both the top-10 accuracy and MRR of the LLMs. The physician performance showed a strong correlation with both top-10 accuracy (ρ=0.565) and MRR (ρ=0.650), with a stronger correlation observed for MRR. Of note, there were cases where a low physician score (2 points) corresponded to a correct diagnosis by the LLM, as well as instances where the physician’s diagnosis was entirely correct while the LLM failed to include it in the top-10 list (Figure 5).

### Prospective Study

#### Physician Performance Evaluation

**Figure 6.**
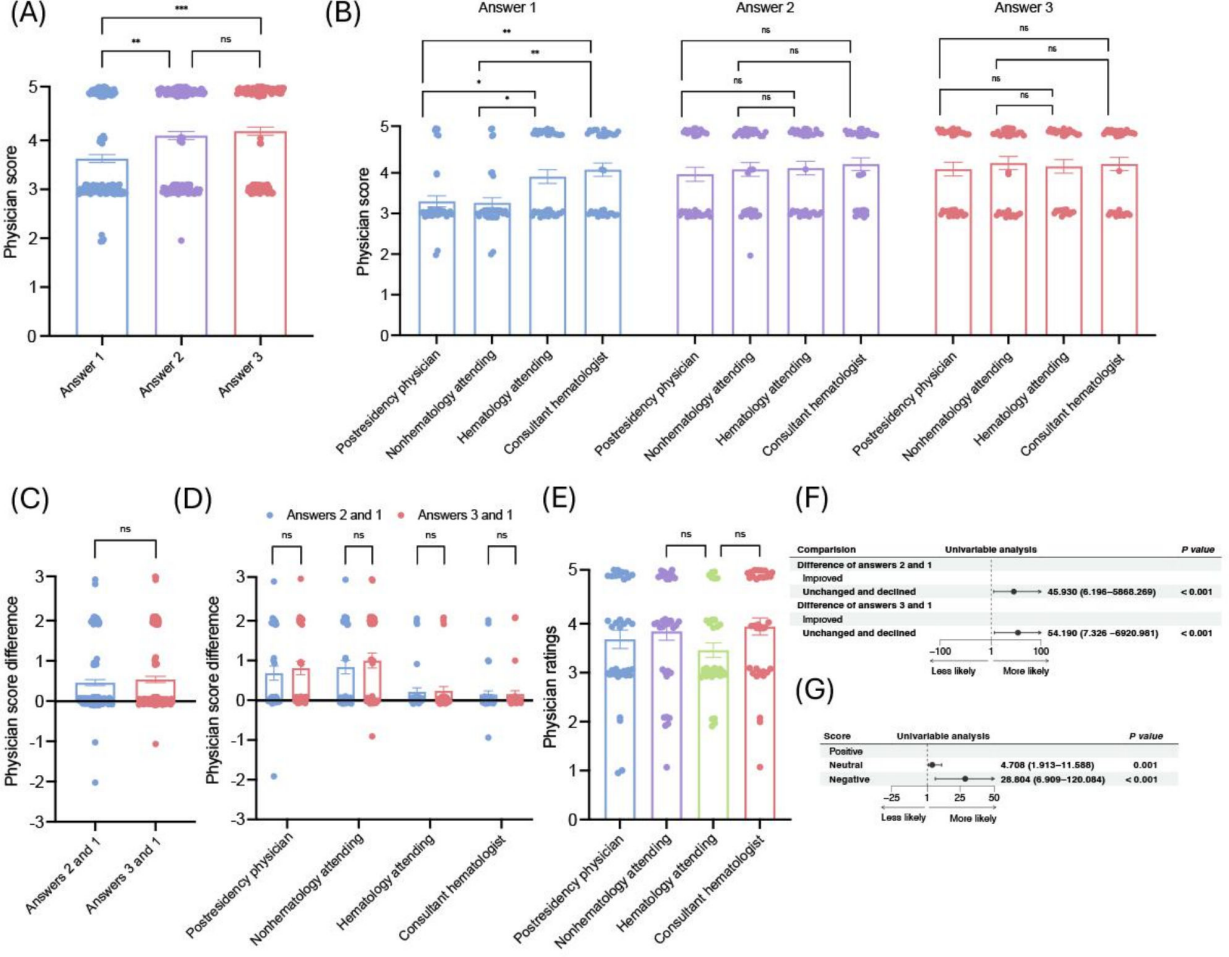
Results of the prospective study. Data points in this figure were jittered for visualization. (A) Scores from all physicians across three answers. (B) Scores by physicians stratified by experience level and answers. (C) Score differences between the second and first answers and between the third and first answers among all physicians. (D) Score differences between the answers 2 and 1 and between answers 3 and 1 by experience level. (E) Subjective ratings of large language model–generated information by physicians with different experience levels. (F) Forest plot showing the impact of biased responses on physician performance improvement. (G) Forest plot showing the impact of biased responses on subjective ratings of LLM-generated information. *P<.05; **P<.01; ***P<.001. ns: not significant.

On the basis of these findings, we conducted a prospective study to investigate whether LLMs can help physicians improve diagnostic performance. In total, 28 physicians from our institution, 7 (25%) at each experience level (postresidency physicians, nonhematology attendings, hematology attendings, and consultant hematologists), were recruited. Each physician independently evaluated all 5 cases in 3 sequential steps as described in the Methods section using an online system. The results showed that, for all participants, diagnostic scores significantly improved on the second attempt (*P*=.006) and further on the third attempt (*P*=.001). However, no significant difference was observed between the second and third attempts (Figure 6A). These findings indicate that the primary benefit to physicians came from the LLM-generated candidate diagnoses themselves. In the first attempt, postresidency physicians differed significantly from both hematology attendings (*P*=.04) and consultant hematologists (*P*=.004). Nonhematology attendings also showed significant differences compared to hematology attendings (*P*=.03) and consultant hematologists (*P*=.002). No significant differences were observed among groups for the second or third attempts (Figure 6B). Thus, while diagnostic performance differed significantly across experience levels in the first attempt, these differences were eliminated following the use of the LLM. Subgroup analysis showed that postresidency physicians improved significantly from the first to both the second and third attempts (*P*=.01 and *P*=.003), and nonhematology attendings showed similar improvements (*P*=.001 and *P*<.001). In contrast, hematology attendings and consultant hematologists did not exhibit significant improvements (Figure S3 in Multimedia Appendix 1), consistent with their already high baseline performance. These findings suggest that LLMs can enhance the diagnostic performance of less-experienced physicians but have less impact on specialists. Across all participants, score improvements from answer 1 to both answers 2 and 3 were not statistically significant (*P*=.17). No significant differences were detected within each physician group (Figure 6C and 6D). Among the 4 physician groups, there were no significant differences in the subjective ratings of information provided by the LLM across group comparisons (Figure 6E), indicating broadly consistent perceptions of utility regardless of seniority.

#### Effects of Biased Responses

Finally, we investigated whether biased responses from the LLM affected physician performance and their AI ratings. When the LLM returned a biased response, physicians’ answers in the second attempt frequently remained unchanged or even declined (odds ratio [OR] 45.930, 95% CI 6.196‐5868.269). Moreover, revealing the CoT and analysis on the third attempt did not reduce this bias effect (OR 54.190, 95% CI 7.326‐6920.981; Figure 6F). Participants also tended to give neutral (OR 4.708, 95% CI 1.913‐11.588) or negative evaluations (OR 28.804, 95% CI 6.909‐120.084) when the LLM’s response was biased (Figure 6G).

## Discussion

### Principal Findings

To our knowledge, this is the first and largest LLM-based diagnostic study on rare hematologic diseases. It is also among the few incorporating a prospective component to assess the effect of LLM-generated information on physician performance. We used real admission records from our hospital information system (none were publicly released), thereby trying to avoid data contamination and enable a more realistic evaluation of LLM performance. Importantly, we departed from conventional approaches that rely on customized prompts, fine-tuning, or strict parameter control. Instead, we used each LLM’s default settings and directly input full admission records, thereby enhancing external validity. We incorporated a prospective phase into our study, applying a stepwise approach to assess how intermediate and final LLM outputs affect physicians with different levels of experience, thereby offering a direct evaluation of their impact on real-world clinical diagnosis. This design was intended to examine the feasibility of integrating mainstream commercial LLMs more rigorously into routine clinical workflows and to capture their practical influence. To our knowledge, some physicians have already begun using similar strategies to support diagnostic decision-making.

In the retrospective phase, our results show that LLMs, particularly new-generation models with CoT reasoning, exhibit strong potential in rare disease diagnosis. In the retrospective phase, ChatGPT-o1-preview achieved 70.3% accuracy, similar to that observed in human physicians. Moreover, it was able to identify rare diagnoses that were initially overlooked by the admitting physicians in some cases. In addition, LLMs demonstrated lower diagnostic accuracy for AL amyloidosis, Castleman disease, ECD, and POEMS syndrome. These conditions are notoriously difficult to diagnose because of multisystem involvement, nonspecific symptoms, and insidious onset, often requiring pathological confirmation or specialized testing. Our quantitative analysis also demonstrated reduced diagnostic keyword density in the admission records for these conditions, which contributes to their greater diagnostic difficulty. In addition, their underrepresentation in the literature limits the available training corpora, thereby reducing the likelihood of effective LLM learning. We also observed an inverse relationship between diagnostic accuracy and output stability across most LLMs. This likely reflects the probabilistic nature of their output. Rare diseases, underrepresented in medical literature and often presented atypically, follow a long-tail distribution: common diseases dominate clinical datasets, whereas the far more numerous rare diseases each occur sparsely and exhibit heterogeneous phenotypes [42]. Consequently, when generating top-k results, models assign similar probabilities to multiple candidates diagnosed with rare diseases, making them highly sensitive to minor perturbations that can induce substantial variability, reduce output stability, and increase diagnostic uncertainty. This effect may be further amplified by CoT reasoning, in which small differences at early steps propagate through subsequent multistep reasoning, ultimately producing divergent diagnoses [43]. Therefore, repeated queries of the same case may yield variable responses. This variability broadens the range of diagnoses, paradoxically increasing the likelihood of identifying the correct rare disease under higher randomness. However, Claude 3.5 Sonnet deviated from this pattern, possibly due to exposure to specific datasets or targeted fine-tuning for medical diagnostic tasks. This suggests that, as with other LLM tasks, architecture, training strategy, model scale, capabilities, and orientation collectively influence the model’s diagnostic performance for rare diseases. However, LLMs may miss correct diagnoses entirely, even when physicians are completely accurate. As reflected by MRR results, correct diagnoses are not always top ranked, requiring careful clinical judgment. When LLM outputs are biased, they often mislead physicians, underscoring the double-edged sword nature of LLMs and the need for critical appraisal and selective integration into clinical workflows, which is consistent with previous findings in common diseases [44]. The indispensable role of physicians still remains clear.

In the prospective phase, postresidency physicians and nonhematology attendings significantly improved their accuracy after reviewing LLM outputs, reaching levels that were not statistically different from those of rare disease specialists at a top-ranked Chinese hospital [45]. However, the third diagnosis made with LLM-generated CoT and analysis did not significantly outperform the second diagnosis. Although average scores slightly increased, this suggests that additional LLM input may not improve diagnostic accuracy in this context. Nonetheless, the rationale provided by LLMs may still hold value in more complex clinical scenarios for physician interpretation and evaluation. Interestingly, hematology attendings appeared to assess LLM-generated information more critically, although the difference was not statistically significant.

Overall, our findings, which include the high diagnostic accuracy observed in the retrospective analysis and the significant improvement in physician performance during the prospective phase, support the practical feasibility of rapidly integrating LLMs into real-world clinical workflows for rare disease diagnosis. In this study, we used mainstream commercial LLMs without fine-tuning and relied exclusively on unimodal text admission records, a design that improves the generalizability and deployability of the approach from a technical integration perspective. Once physicians complete the admission notes, the LLM can process the input and provide diagnostic suggestions through a workflow closely aligned with conventional clinical decision support systems (CDSS), which are already widely adopted in clinical practice. Compared with CDSS, LLMs may provide specific advantages in the context of rare diseases. Traditional CDSS perform effectively in specific and well-defined tasks due to their rule-based logic and interpretability. They have been extensively applied in areas such as medication review, sepsis alerts, and risk prediction for deep vein thrombosis and pressure ulcers, including those designed to assist diagnosis [46,47]. However, these systems are often trained based on structured and predefined clinical data, which restricts adaptability and increases the cost of maintenance and updates. This limitation is particularly evident in rare diseases, where data scarcity and phenotypic heterogeneity present major challenges. A review of traditional CDSS in rare diseases has noted that many systems are no longer actively maintained, and only a few remain available for testing, most likely for these reasons [48]. In contrast, LLMs can directly process unstructured free-text input and generate multiple candidate diagnoses. They can now also provide stepwise reasoning through CoT mechanisms. When combined with online search strategies or iterative model updates, LLMs can effectively incorporate more information, such as newly published case reports and guidelines, which is especially valuable in rare disease contexts. Their user-friendly interface further improves accessibility and deployment. Despite these advantages, challenges still remain in certain areas. The black box nature and hallucination issues of LLMs remain prominent. Compared with CDSS, they introduce indeterminacy, greater bias risks, and limited explainability even with CoT, which may intensify patient concerns regarding the use of LLMs by clinicians [49]. These concerns highlight the need for rigorous safeguards to ensure responsible integration into clinical practice [50].

Therefore, both the double-edged sword effect demonstrated in our findings and the inherent characteristics of LLMs in clinical translation highlight the need for multiple safeguards. One approach is to apply confidence thresholds and selectively present outputs. When the model’s predicted confidence falls below a predefined threshold, the corresponding result may be withheld [51,52]. In such cases, the system can enforce the requirement for additional diagnostic information or direct the case to a manual verification process. Another approach is uncertainty labeling, in which the model’s confidence is conveyed in various forms, such as brief textual indications of uncertainty (eg, “I am not sure” and “I don’t know”) or probability intervals, thereby guiding user expectations [53]. Combining outputs from multiple models or repeated runs of a single model (ie, self-consistency) may further reduce diagnostic errors [54-56]. In addition, developing standardized hybrid human-AI systems may be particularly beneficial when discrepancies arise between LLM outputs and physician judgment [57]. Previous studies have demonstrated that these measures can effectively mitigate bias. This should also be reflected in the educational process.

From the perspective of physician education in AI use, physicians must be trained to critically evaluate LLM suggestions by identifying unsupported inferences, verifying them against guidelines or other independent sources, and determining when to reject model outputs. Although we cited a case where a patient used an LLM for self-diagnosis, his history had already been recorded by professionals. We do not recommend self-diagnosis by nonprofessionals, as untrained individuals often fail to objectively report their medical history or physical findings and seek only a single definitive diagnosis without differential diagnosis, potentially leading to unnecessary anxiety and mistrust in health care. This is an essential concept that the medical community must actively communicate to the public. More importantly, our prospective results further demonstrated that LLMs can improve diagnostic accuracy among less-experienced clinicians, underscoring their educational value. We believe this is particularly applicable to problem-based learning sessions during residency and early-stage continuing medical education. Under faculty supervision, trainees can use LLM-generated candidate diagnoses to analyze the model’s reasoning process and clarify distinctions among related conditions. This approach is practical, preserves educational value, and reduces the risk of bias from LLMs.

### Limitations

This study has several limitations. First, both the retrospective and prospective analyses were conducted at a single center. Despite a previous power analysis, the study is limited by the relatively small sample size, including the number of disease types, case records, and participants in the prospective phase, as well as the use of a single language, which may affect external validity. Large multicenter and international studies are warranted to further validate these findings. Second, relying solely on single-modality text data limited the LLM’s potential to incorporate multimodal information such as imaging and comprehensive laboratory results, which might enhance diagnostic accuracy for rare diseases. We used only admission records, as outpatient notes in Chinese tertiary hospitals are typically brief and lack detail, with physicians often seeing nearly 100 patients per day. Therefore, certain rare diseases primarily diagnosed in outpatient settings were underrepresented. The absence of a pediatric department in our center also limited inclusion of genetic hematologic disorders such as congenital hemophilia. Despite our extensive efforts to prevent data leakage and ensure deidentification, certain risks remain. Although the medical records used were not publicly available, patients may have independently shared their data online, and we cannot guarantee that such data were entirely excluded from LLM training. Meanwhile, although all medical records were deidentified before analysis, the risk of reidentification cannot be entirely eliminated, particularly in rare diseases where combinations of age, clinical features, and treatment history may be unique. To mitigate this, 2 physicians manually redacted direct identifiers (eg, names, contacts, record numbers, and locations), and inputs were restricted to key clinical findings. To ensure a rigorous standard for evaluating LLMs, we defined a correct diagnosis as one that explicitly identified the disease. However, a different standard was applied to human physicians, who often use cautious admission diagnoses for clinical safety. For instance, in one case of LCH, the admission diagnosis was “left scapular lesion under investigation,” yet the initial note clearly considered LCH. To address potential underestimation of physician reasoning, we implemented a 5-point rating system. Moreover, unlike LLMs, human physicians rarely generate 10 differential diagnoses, placing them at a structural disadvantage in head-to-head comparisons. Because multiple factors could either under- or overestimate performance for both LLMs and physicians, we refrained from direct accuracy comparisons.

### Conclusions

Without any task-specific fine-tuning, publicly available new-generation LLMs can use text-only admission records to suggest correct diagnoses for rare hematologic diseases with diagnostic accuracy similar to that of human physicians. In a prospective evaluation, providing LLM-generated differential diagnoses significantly enhanced the diagnostic performance of less-experienced physicians. However, no improvement was observed among specialists. LLMs that produce biased responses markedly diminish these performance gains, and this deficit cannot be remedied by additional CoT or further analysis. Thus, LLMs serve as a double-edged sword in rare hematologic disease diagnosis and must be applied with caution and appropriate safeguard systems to ensure that medical professionals maintain independent clinical judgment.

## Supplementary material

10.2196/77334 Multimedia Appendix 1 Supplementary data with detailed methods, extended statistical results, and additional figures and tables supporting the findings of this study.

## Abbreviations

AI: artificial intelligence
AL: amyloid light-chain
CDSS: clinical decision support systems
CoT: chain-of-thought
ECD: Erdheim-Chester disease
LCH: Langerhans cell histiocytosis
LLM: large language model
MRR: mean reciprocal rank
OR: odds ratio
POEMS: polyneuropathy, organomegaly, endocrinopathy, monoclonal gammopathy, and skin changes
