# Supplementary material for: Performance of Large Language Models in Diagnosing Rare Hematologic Diseases and the Impact of Their Diagnostic Outputs on Physicians: Combined Retrospective and Prospective Study
Source: J Med Internet Res. 2025 Oct 9;27:e77334. doi: 10.2196/77334 (PMC12511990; doi:10.2196/77334)
Supplement: Multimedia Appendix 1 [file jmir-v27-e77334-s001.docx]

**Supplementary Methods**

*Input data collection*

The study was conducted in accordance with the Declaration of Helsinki, with approval from the Institutional Review Board of West China Hospital, Sichuan University (approval number: 2024-1754), and registration in the Chinese Clinical Trial Registry (ChiCTR2400089959). We selected all 25 rare hematologic diseases included in the first and second lists of rare diseases in the People’s Republic of China for screening (1, 2). These 25 diseases also meet rare disease criteria in the United States and Europe (3, 4). We searched our hospital information system for all inpatients who once had primary diagnosis of any of these diseases. In accordance with China’s previous rare disease information management regulations, a maximum of 20 patient records were included for each disease (5). Admission records were identified in reverse chronological order without additional manual filtering beyond the exclusion criteria, even if there were significant discrepancies between the initial diagnosis and final diagnosis upon retrospective review, to minimize manual selection and maximize sample representativeness. For each patient, only the first hospitalization related to the rare disease was included, regardless of whether the diagnosis was confirmed at discharge. Admissions with definitive diagnostic evidence (e.g., positive Congo red staining for AL amyloidosis) and those for day-case chemotherapy or surgery were excluded. If fewer than eight eligible records were found for a given disease, that disease was excluded from the study to ensure representativeness. All included records were re-verified according to standard diagnostic guidelines and consensus criteria (6-14).

*Input data processing*

For records meeting the inclusion criteria, we manually removed all diagnostically conclusive content, including admitting, imaging, and pathological diagnoses. Descriptive findings from imaging or pathology reports were retained (e.g., for AL amyloidosis, late gadolinium enhancement on cardiac magnetic resonance imaging or immunofluorescence staining of pathological tissue demonstrating light-chain restriction). We also ensured the removal of all potentially identifiable personal information. This step was independently performed and cross-validated by two physicians (HY and TC) to guarantee completeness. The processed records were compiled into a standardized table in their original Chinese, without translation, for subsequent LLM analysis.

*LLM analysis*

We employed seven mainstream LLMs for the analyses: Claude 3.5 Sonnet (claude-3-5-sonnet-20241022), DeepSeek-R1 (deepseek-reasoner), Doubao (Doubao-1.5-Pro-256k), Gemini Experimental 1206 (gemini-exp-1206), ChatGPT 4o (gpt-4o-2024-11-20), ChatGPT o1-preview (o1-preview-2024-09-12), and Qwen (Qwen-Max-2025-01-25). Access to these models was obtained through their official application programming interfaces (APIs) in Python, retrieving the 10 most likely diagnoses for each medical record in five repeated responses. The parameters temperature, top-p, seed, and max tokens were not controlled and remained at their default settings. All models were prompted in Chinese as follows (English translation provided):

“Please assume you are an experienced professional physician. Here is an admission medical record. Based on its content, provide the 10 most likely main diagnoses, ranked from most to least probable.”

Two physicians (HY and TC) inspected the generated results to ensure they were valid and non-refusal responses. We then used the gpt-4o-2024-11-20 model, set as "professional medical expert and data processing assistant" role with temperature = 0, to standardize and merge identical or similar diagnoses for each patient record (e.g., "chronic kidney disease," "chronic renal insufficiency," and "stage 4 chronic kidney disease" were all unified under "chronic kidney disease"). The prompt was as follows (English translation provided):

“Please help me merge the following diagnoses that are identical, similar, or close in meaning into one standardized diagnostic name, following these rules:

1. If a diagnosis name contains parentheses, use the content within the parentheses as the standard name. For example, from ‘Rheumatic disease (e.g., rheumatoid arthritis),’ extract ‘rheumatoid arthritis’ as the standard name.
2. The standard name should not include parentheses or extraneous explanations; keep only the diagnosis itself.”

The merged diagnoses were subsequently reviewed by two physicians (HY and TC) to ensure no incorrect merges. Then, the merged diagnoses were manually checked for correctness. If a correct diagnosis was identified among the list, we standardized its name for subsequent statistical analyses. The “correct” diagnosis had to capture the essential features of the disease. For example, “systemic light-chain amyloidosis,” “amyloid cardiomyopathy,” “amyloid nephropathy,” and “light-chain deposition disease” were all accepted as representing “AL amyloidosis,” whereas “plasma cell disease” was not. For Castleman disease, general terms such as “lymphoproliferative disorder” were deemed incorrect. In the case of acquired hemophilia, the diagnosis had to specify its acquired nature (e.g., “acquired hemophilia” or “acquired coagulation factor deficiency”); “hemophilia” alone was insufficient. All judgments were independently cross validated by two physicians (HY and TC).

*Human physician diagnoses processing*

To evaluate both the original admission diagnoses and the subsequent prospective diagnoses made by human physicians, we adopted a 5-point rating system: 5 points for a completely correct diagnosis, 4 points if the diagnosis was in the correct direction, 3 points if there was no diagnostic error and the description primarily outlined symptoms objectively, 2 points if the diagnosis demonstrated an incorrect tendency, and 1 point for a completely incorrect diagnosis (Supplementary Table 1). Admitting diagnoses recorded in the admission notes were evaluated. HY and TC independently scored each case. Concordant scores were accepted, and in cases of disagreement, the final score was determined by a third physician (YW).

*Statistical methods for the retrospective data*

We calculated the Top-10 accuracy (Top-10) for each model to evaluate whether the LLM could provide at least one correct diagnosis among the top 10 returned diagnoses. If at least one correct diagnosis 𝐷 was present within the top 10 diagnoses, it was recorded as 1; otherwise, it was recorded as 0. For each medical record, we conducted five repeated tests and computed the average Top-10 accuracy. The calculation formula for one medical record is as Equation (1): $\begin{aligned} Top-10=\frac{D_{1} + D_{2} + D_{3} + D_{4} + D_{5}}{5}\#\text{(1)} \end{aligned}$

Given that the ranking of diagnoses generated by the LLM is also crucial for evaluating its accuracy, we further employed the mean reciprocal rank (MRR) as a performance metric. For each patient record, the model generated five repeated responses. In each response, if the correct diagnosis appeared at rank r_i_ (i.e., its position in the response list), the reciprocal rank for that set was calculated as the reciprocal of r_i_. If the correct diagnosis was not present in the response, the reciprocal rank for that response was assigned a value of 0. The MRR for one medical record was then computed as Equation (2):

$$\begin{aligned} MRR=\frac{1}{5}\sum_{i = 1}^{5} \frac{1}{r_{i}}\#\text{(2)} \end{aligned}$$

Diagnostic keyword density was calculated using a weighted scoring system, assigning 5 points to diagnosis-specific keywords and 1 point to diagnosis-supportive keywords, with repeated occurrences of similar keywords counted only once. All regular expressions and source code are publicly available on GitHub.

To evaluate the similarity of diagnosis lists provided by the same model across repeated responses for one medical record, we used the **Jaccard similarity** as a metric. For each diagnosis d in a single patient record across five repeated responses, the Jaccard similarity for one medical record was computed as Equation (3):

$$\begin{aligned} Jaccard similarity=\frac{\left| \bigcap_{i=1}^{5} d_{i} \right|}{\left| \bigcup_{i=1}^{5} d_{i} \right|}\#\left( 3 \right) \end{aligned}$$

To evaluate the ranking stability of diagnoses, we used entropy as defined in Equation (4). The probability p_i_ of a diagnosis appearing at rank 𝑖 was computed as the ratio of its frequency at that rank to its total occurrences in all Top-10 rankings (Equation (5)). If a diagnosis did not appear in a given rank, we assigned an equal probability across all ranks, as shown in Equation (6), to prevent numerical instability.

$$\begin{aligned} entropy=-\sum_{i=1}^{10} p_{i}\log p_{i}\#\left( 4 \right) \end{aligned}$$

$$\begin{aligned} p_{i}=\frac{\text{count}\left( d_{i} \right)}{\sum_{k=1}^{10} \text{count}\left( d_{k} \right)}\#\left( 5 \right) \end{aligned}$$

$$\begin{aligned} p_{i}=\frac{1}{10}\#\left( 6 \right) \end{aligned}$$

To compare across groups, one-way analysis of variance (ANOVA) was applied. We used Spearman’s rank correlation coefficient (ρ) to assess the association between doctor scores and both the Top-10 diagnostic list and MRR.

*Study design for the prospective phase*

To evaluate how LLM-generated results affect human physicians’ diagnostic performance, we developed an online questionnaire using the Wenjuanxing web platform. From the admission records used in this study for LLM-based retrospective diagnosis, we randomly selected five records covering different diseases. In February 2025, these records were analyzed by the ChatGPT o1 model via its web interface using the same prompts but requiring additional analysis employed for the retrospective API. The prompt was as follows (English translation provided):

“Please assume you are an experienced professional physician. Here is an admission medical record. Based on its content, provide the 10 most likely main diagnoses, ranked from most to least probable with analysis.”

We saved the generated CoT and analysis content (translating English response into Chinese via ChatGPT 4o when needed).

To determine the sample size, we conducted a power analysis using G*Power (Version 3.1.9.7). Under the settings of F tests, ANOVA: Repeated measures, within-between interaction, and a priori analysis, we specified an effect size (f) of 0.25, α error probability of 0.05, power of 0.8, correlation among repeated measures of 0.7, and a nonsphericity correction ε of 1. Assuming one question per participant in a 4-group, 3-timepoint design, the calculated total sample size was 28, which we subsequently enrolled.

In March 2025, after informing participants that the questionnaire aimed to “diagnose five diseases based on admission records,” we recruited four physician groups from West China Hospital of Sichuan University: post-residency physicians, non-hematology attendings, hematology attendings, and consultant hematologists. Participants were randomly assigned one questionnaire set and received 100 Chinese Yuan upon completion. To minimize potential bias, TC independently designed the questionnaires, and HY independently distributed them. The online questionnaire was designed as follows: for each case, the first page presented the full admission record and asked physicians to provide the primary diagnosis and differential diagnoses. The second page included the same admission record along with the top 10 diagnoses generated by ChatGPT o1, requiring physicians to reassess and provide their diagnoses again. The third page additionally presented the model-generated CoT reasoning and analytical content, and physicians were required to reassess the case and provide their diagnoses for the third and final time. On the fourth page, the correct diagnosis was revealed, and physicians were asked to rate the information provided by the LLM. We adopted a 5-point rating system for subjective rating. The questionnaire structure and full rating criteria are provided in the supplementary materials (Supplementary Table 2 and 3). Each questionnaire set consisted of five clinical cases. Once initiated, physicians were not allowed to revisit previous pages.

*Statistical methods for the prospective data*

To compare physicians’ performance across groups, we applied Wilcoxon matched-pairs signed rank test, Kruskal-Wallis test, or nonparametric longitudinal data analysis based on data. To analyze the impact of LLM generated diagnoses on improvement in physicians’ performance, we defined responses in which ChatGPT o1 failed to produce the correct diagnosis as biased response. An increase from the first to the second or third answers was classified as performance improved; no change or a decrease was defined as unchanged or declined. Subjective ratings of 4–5 were considered positive, 3 as neutral, and 1–2 as negative. Firth logistic regression was then used to address data separation issues and calculated ORs, 95% CI and *P* values. For subjective rating analyses that violated the Brant test assumption, we used a nominal multinomial regression model, reporting ORs, 95% CI and *P* values using the Wald method. All statistical analyses and visualizations were conducted using Python (Version 3.13.1), R (Version 4.4.2), and GraphPad Prism (Version 10.4).

*Data Availability Statement*

The code associated with this project is available on GitHub: https://github.com/glamic/hemararediseases_llmcompare

**References**

1. The Government of the People's Republic of China. Notice on the Publication of the First List of Rare Diseases 2018 [20/03/2025]. Available from: <https://www.gov.cn/zhengce/zhengceku/2018-12/31/content_5435167.htm>.

2. The Government of the People's Republic of China. Notice on the Publication of the Second List of Rare Diseases 2023 [20/03/2025]. Available from: <https://www.gov.cn/zhengce/zhengceku/202309/content_6905273.htm>.

3. U.S. Food & Drug Administration. Rare Diseases at FDA 2024 [20/03/2025]. Available from: <https://www.fda.gov/patients/rare-diseases-fda>.

4. European Commission. Rare diseases[cited 2025 20/03/2025]. Available from: <https://health.ec.europa.eu/rare-diseases-and-european-reference-networks/rare-diseases_en#latest-updates-and-documents>.

5. Ministry of Science and Technology of the People's Republic of China. Notice of the Ministry of Science and Technology on Soliciting Public Opinions on the 'Detailed Rules for the Implementation of the Regulations on the Administration of Human Genetic Resources (Draft for Solicitation of Comments) 2022 [cited 2025 20/03/2025].

6. ​Chinese Collaborative Group for Systemic Light Chain Amyloidosis, National Clinical Research Center for Kidney Diseases, ​National Clinical Research Center for Hematologic Diseases. Guidelines for the Diagnosis and Treatment of Systemic Light Chain Amyloidosis (2021 Revision). Zhonghua Yi Xue Za Zhi. 2021;101(22):1646-56.

7. Hematology Committee of Chinese Medical Association, Hematological Oncology Committee of China Anti-Cancer Association, China Castleman Disease Network (CCDN). The consensus of the diagnosis and treatment of Castleman disease in China (2021). Zhonghua Xue Ye Xue Za Zhi. 2021;42(7):529-34.

8. Estrada-Veras JI, O’Brien KJ, Boyd LC, Dave RH, Durham BH, Xi L, et al. The clinical spectrum of Erdheim-Chester disease: an observational cohort study. Blood Advances. 2017;1(6):357-66.

9. Dispenzieri A. POEMS Syndrome: 2019 Update on diagnosis, risk-stratification, and management. American Journal of Hematology. 2019;94(7):812-27.

10. Hematology Oncology Committee of China Anti-Cancer Association, Chinese Society of Hematology, Chinese Medical Association, Chinese Working Group of Walderström Macroglobulinemia. Chinese guideline for diagnosis and treatment of lymphoplasmacytic lymphoma/Waldenström macroglobulinemia (2022). Zhonghua Xue Ye Xue Za Zhi. 2022-08-14;43(08).

11. Thrombosis and Hemostasis Group, Chinese Society of Hematology, Chinese Medical Association, Hemophilia Treatment Center Collaborative Network of China. Chinese guidelines on the diagnosis and treatment of acquired hemophilia A (2021). Zhonghua Xue Ye Xue Za Zhi. 2021-10-14;42(10).

12. Goyal G, Tazi A, Go RS, Rech KL, Picarsic JL, Vassallo R, et al. International expert consensus recommendations for the diagnosis and treatment of Langerhans cell histiocytosis in adults. Blood. 2022;139(17):2601-21.

13. Lymphoma Expert Committee of Chinese Society of Clinical Oncology (CSCO). Chinese expert consensus on common primary cutaneous T-cell lymphoma (2024 version). Journal of Leukemia & Lymphoma. 2024-06-25;33(06).

14. Thrombosis and Hemostasis Group Chinese Society of Hematology, Chinese Medical Association. Chinese guideline on the diagnosis and management of thrombotic thrombocytopenic purpura (2022). Zhonghua Xue Ye Xue Za Zhi. 2022-01-14;43(01).

**Figure S1.** PubMed search results quantifying the number of publications for each disease based on standardized disease names (conducted on 2025-09-10). For ChatGPT o1-preview-2024-09-12 and deepseek-reasoner, the ranking of diagnostic accuracy was exactly concordant with publication volume for four more difficult-to-diagnose diseases, suggesting a potential link between corpus richness and LLM diagnostic capability in rare diseases.

**Figure S2.** Diagnostic keyword density across diseases, defined as the number of strongly disease-related keywords per 1,000 Chinese characters in admission records. AL amyloidosis (2.27), Castleman disease (2.55), ECD (2.28), and POEMS syndrome (3.53) exhibited markedly lower densities compared with WM (4.81), acquired hemophilia (6.76), TTP (7.24), LCH (4.81), and CTCL (8.53).

**
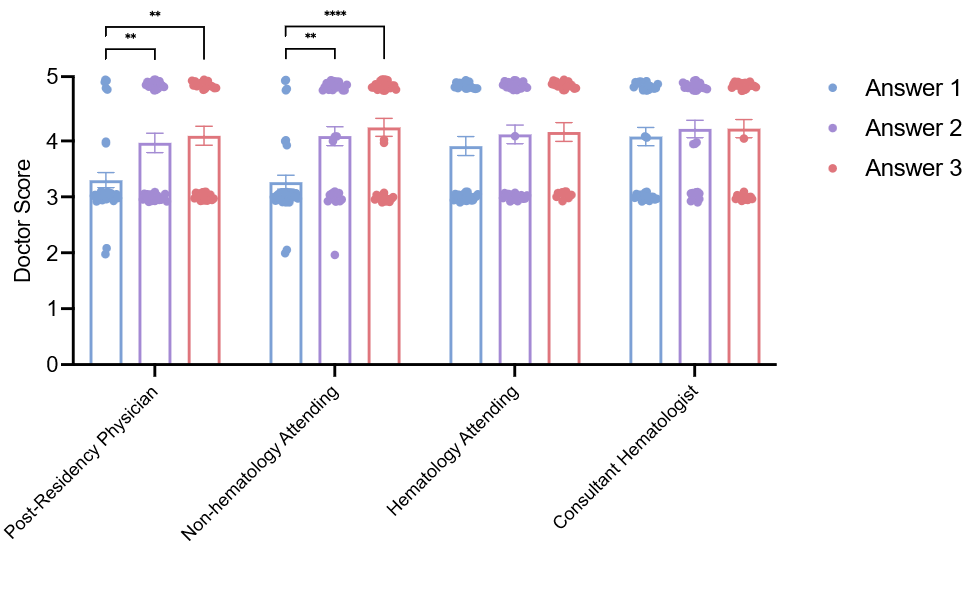
**

**Figure S3.** Scores by physicians stratified by answers and experience level. Data points in this figure were jittered for visualization.

**Table S1.** A 5-point rating system for evaluating both the original admission diagnoses and the prospective diagnoses made by human physicians.

| **Score** | **Explanation** |
| --- | --- |
| 5 points  (completely correct diagnosis) | The diagnosis was entirely correct. |
| 4 points  (generally correct reasoning) | The reasoning was in the correct direction, aiding in an accurate diagnosis. |
| 3 points  (no diagnostic error) | No clear mistakes were present; the diagnosis primarily described symptoms objectively. |
| 2 points  (questionable reasoning) | The reasoning showed an incorrect tendency, making a correct diagnosis unlikely. |
| 1 point  (completely incorrect diagnosis) | The diagnosis was entirely wrong. |

**Table S2.** The online questionnaire structure for prospective study.

| **Introductory Page** |
| --- |
| Below are admission records for 5 patients. Please provide your primary diagnosis and differential diagnoses based on the following medical records. Try to list as many differential diagnoses as possible. There is no required format for the diagnoses—just present your diagnostic reasoning. Examples are provided below:  Example 1:  Anemia (Iron deficiency? Chronic inflammation?)  Example 2:  Myelodysplastic syndrome? Aplastic anemia?  Example 3:  Peripheral nerve damage (Immune-related? Paraneoplastic syndrome?) |
| **Case N, Page 1** |
| [Medical record]  Please provide your diagnosis. |
| **Case N, Page 2** |
| We have additionally included the AI’s diagnostic result for this case. Please provide your diagnosis again.  Please provide your diagnosis.  [Medical record]  [LLM Top-10 diagnosis result] |
| **Case N, Page 3** |
| We have additionally included the AI’s diagnostic result, its reasoning process and analysis. Please provide your diagnosis again.  [Medical record]  [LLM Top-10 diagnosis result, chain of thought and analysis.] |
| **Case N, Page 4** |
| The patient’s final diagnosis was: [Correct Disease]. Please rate the reasoning process generated by the AI for this case.  [Rating system, Supplementary Table 3] |

**Table S3.** A 5-point rating system for evaluating the information provided by the LLM.

| **Score** | **Explanation** |
| --- | --- |
| **5 Points** | AI diagnosis is extremely valuable, significantly facilitating accurate clinical decision-making.   - The key information provided by AI directly leads to the correct diagnosis. - The AI’s reasoning is exceptional, taking into account all crucial factors including patient history, examination results, and imaging data. - Its proposed differential diagnosis is highly insightful, with possibilities ranked in a proper and logically sound manner. |
| **4 Points** | **AI diagnosis provides significant assistance, although the doctor still needs to supplement judgment.**   - The AI’s overall diagnostic direction is essentially correct, yet clinicians must integrate their own experience to adjust or refine it. - The reasoning is strong, though a few key etiologies might not be adequately prioritized. - The AI’s differential diagnosis is valuable, although the ranking and probability assessments could be further refined. |
| **3** Points | AI diagnosis has some reference value but does not substantially impact the final clinical judgment.   - The AI identifies several potential diagnoses but does not significantly influence the physician’s overall diagnosis. - The reasoning is generally sound, albeit with the potential to overlook some important details. - The AI’s differential diagnosis provides certain useful directions, although the ordering or weighting of possibilities may be suboptimal. |
| **2 Points** | **The AI diagnosis shows considerable deviation and may even mislead the doctor.**   - The primary diagnostic direction suggested by the AI could be incorrect or insufficiently relevant. - The reasoning appears biased, with the AI possibly over-relying on partial data while neglecting patient history or critical clinical signs. - Its differential diagnosis is inappropriately broad, omitting some of the more likely etiologies. |
| **1 Points** | **The AI diagnosis is entirely unhelpful and may hinder the doctor’s judgment.**   - The diagnostic direction provided by the AI is completely wrong, and reliance on it could lead to misdiagnosis. - The reasoning is poor, overly vague, or lacking clinical relevance, possibly even contradicting established medical knowledge. - Its differential diagnosis is unreasonable, with the AI overemphasizing irrelevant factors while neglecting the patient’s primary symptoms. |

**Table S4.** Non-parametric one-way ANOVA to test the difference in Top-10 accuracy among different LLMs

| **Dunn's multiple comparisons test** | **Mean rank diff.** | **Summary** | **Adjusted P Value** |
| --- | --- | --- | --- |
| claude-3-5-sonnet-20241022 vs. deepseek-reasoner | -95.64 | ns | 0.068 |
| claude-3-5-sonnet-20241022 vs. Doubao-1.5-Pro-256k | 217.9 | **** | <0.0001 |
| claude-3-5-sonnet-20241022 vs. Gemini Experimental 1206 | -26.85 | ns | >0.9999 |
| claude-3-5-sonnet-20241022 vs. gpt-4o-2024-11-20 | 20.1 | ns | >0.9999 |
| claude-3-5-sonnet-20241022 vs. o1-preview-2024-09-12 | -161.1 | **** | <0.0001 |
| claude-3-5-sonnet-20241022 vs. Qwen-Max-2025-01-25 | 92.92 | ns | 0.0888 |
| deepseek-reasoner vs. Doubao-1.5-Pro-256k | 313.5 | **** | <0.0001 |
| deepseek-reasoner vs. Gemini Experimental 1206 | 68.78 | ns | 0.7184 |
| deepseek-reasoner vs. gpt-4o-2024-11-20 | 115.7 | ** | 0.0077 |
| deepseek-reasoner vs. o1-preview-2024-09-12 | -65.48 | ns | 0.9199 |
| deepseek-reasoner vs. Qwen-Max-2025-01-25 | 188.6 | **** | <0.0001 |
| Doubao-1.5-Pro-256k vs. Gemini Experimental 1206 | -244.7 | **** | <0.0001 |
| Doubao-1.5-Pro-256k vs. gpt-4o-2024-11-20 | -197.8 | **** | <0.0001 |
| Doubao-1.5-Pro-256k vs. o1-preview-2024-09-12 | -379 | **** | <0.0001 |
| Doubao-1.5-Pro-256k vs. Qwen-Max-2025-01-25 | -125 | ** | 0.0025 |
| Gemini Experimental 1206 vs. gpt-4o-2024-11-20 | 46.95 | ns | >0.9999 |
| Gemini Experimental 1206 vs. o1-preview-2024-09-12 | -134.3 | *** | 0.0008 |
| Gemini Experimental 1206 vs. Qwen-Max-2025-01-25 | 119.8 | ** | 0.0048 |
| gpt-4o-2024-11-20 vs. o1-preview-2024-09-12 | -181.2 | **** | <0.0001 |
| gpt-4o-2024-11-20 vs. Qwen-Max-2025-01-25 | 72.82 | ns | 0.5246 |
| o1-preview-2024-09-12 vs. Qwen-Max-2025-01-25 | 254 | **** | <0.0001 |

**Table S5.** Non-parametric one-way ANOVA to test the difference in MRR among different LLMs

| **Dunn's multiple comparisons test** | **Mean rank diff.** | **Summary** | **Adjusted P Value** |
| --- | --- | --- | --- |
| claude-3-5-sonnet-20241022 vs. deepseek-reasoner | -77.82 | ns | 0.3925 |
| claude-3-5-sonnet-20241022 vs. Doubao-1.5-Pro-256k | 217 | **** | <0.0001 |
| claude-3-5-sonnet-20241022 vs. Gemini Experimental 1206 | -24.23 | ns | >0.9999 |
| claude-3-5-sonnet-20241022 vs. gpt-4o-2024-11-20 | 22.35 | ns | >0.9999 |
| claude-3-5-sonnet-20241022 vs. o1-preview-2024-09-12 | -147.9 | *** | 0.0002 |
| claude-3-5-sonnet-20241022 vs. Qwen-Max-2025-01-25 | 94.02 | ns | 0.0944 |
| deepseek-reasoner vs. Doubao-1.5-Pro-256k | 294.8 | **** | <0.0001 |
| deepseek-reasoner vs. Gemini Experimental 1206 | 53.59 | ns | >0.9999 |
| deepseek-reasoner vs. gpt-4o-2024-11-20 | 100.2 | ns | 0.0519 |
| deepseek-reasoner vs. o1-preview-2024-09-12 | -70.08 | ns | 0.718 |
| deepseek-reasoner vs. Qwen-Max-2025-01-25 | 171.8 | **** | <0.0001 |
| Doubao-1.5-Pro-256k vs. Gemini Experimental 1206 | -241.3 | **** | <0.0001 |
| Doubao-1.5-Pro-256k vs. gpt-4o-2024-11-20 | -194.7 | **** | <0.0001 |
| Doubao-1.5-Pro-256k vs. o1-preview-2024-09-12 | -364.9 | **** | <0.0001 |
| Doubao-1.5-Pro-256k vs. Qwen-Max-2025-01-25 | -123 | ** | 0.0042 |
| Gemini Experimental 1206 vs. gpt-4o-2024-11-20 | 46.58 | ns | >0.9999 |
| Gemini Experimental 1206 vs. o1-preview-2024-09-12 | -123.7 | ** | 0.0039 |
| Gemini Experimental 1206 vs. Qwen-Max-2025-01-25 | 118.2 | ** | 0.0074 |
| gpt-4o-2024-11-20 vs. o1-preview-2024-09-12 | -170.2 | **** | <0.0001 |
| gpt-4o-2024-11-20 vs. Qwen-Max-2025-01-25 | 71.67 | ns | 0.6368 |
| o1-preview-2024-09-12 vs. Qwen-Max-2025-01-25 | 241.9 | **** | <0.0001 |

**Table S6.** Non-parametric one-way ANOVA to test the difference in Jaccard similarity among different LLMs

| **Dunn's multiple comparisons test** | **Mean rank diff.** | **Summary** | **Adjusted P Value** |
| --- | --- | --- | --- |
| claude-3-5-sonnet-20241022 vs. deepseek-reasoner | 585.5 | **** | <0.0001 |
| claude-3-5-sonnet-20241022 vs. Doubao-1.5-Pro-256k | 120.4 | * | 0.0168 |
| claude-3-5-sonnet-20241022 vs. Gemini Experimental 1206 | 423.5 | **** | <0.0001 |
| claude-3-5-sonnet-20241022 vs. gpt-4o-2024-11-20 | 646.4 | **** | <0.0001 |
| claude-3-5-sonnet-20241022 vs. o1-preview-2024-09-12 | 557.6 | **** | <0.0001 |
| claude-3-5-sonnet-20241022 vs. Qwen-Max-2025-01-25 | 253.6 | **** | <0.0001 |
| deepseek-reasoner vs. Doubao-1.5-Pro-256k | -465.1 | **** | <0.0001 |
| deepseek-reasoner vs. Gemini Experimental 1206 | -162.1 | *** | 0.0001 |
| deepseek-reasoner vs. gpt-4o-2024-11-20 | 60.82 | ns | >0.9999 |
| deepseek-reasoner vs. o1-preview-2024-09-12 | -27.98 | ns | >0.9999 |
| deepseek-reasoner vs. Qwen-Max-2025-01-25 | -331.9 | **** | <0.0001 |
| Doubao-1.5-Pro-256k vs. Gemini Experimental 1206 | 303 | **** | <0.0001 |
| Doubao-1.5-Pro-256k vs. gpt-4o-2024-11-20 | 525.9 | **** | <0.0001 |
| Doubao-1.5-Pro-256k vs. o1-preview-2024-09-12 | 437.1 | **** | <0.0001 |
| Doubao-1.5-Pro-256k vs. Qwen-Max-2025-01-25 | 133.2 | ** | 0.0044 |
| Gemini Experimental 1206 vs. gpt-4o-2024-11-20 | 222.9 | **** | <0.0001 |
| Gemini Experimental 1206 vs. o1-preview-2024-09-12 | 134.1 | ** | 0.004 |
| Gemini Experimental 1206 vs. Qwen-Max-2025-01-25 | -169.9 | **** | <0.0001 |
| gpt-4o-2024-11-20 vs. o1-preview-2024-09-12 | -88.81 | ns | 0.2823 |
| gpt-4o-2024-11-20 vs. Qwen-Max-2025-01-25 | -392.7 | **** | <0.0001 |
| o1-preview-2024-09-12 vs. Qwen-Max-2025-01-25 | -303.9 | **** | <0.0001 |

**Table S7.** Non-parametric one-way ANOVA to test the difference in entropy among different LLMs

| **Dunn's multiple comparisons test** | **Mean rank diff.** | **Summary** | **Adjusted P Value** |
| --- | --- | --- | --- |
| claude-3-5-sonnet-20241022 vs. deepseek-reasoner | -659.1 | **** | <0.0001 |
| claude-3-5-sonnet-20241022 vs. Doubao-1.5-Pro-256k | -154.2 | *** | 0.0004 |
| claude-3-5-sonnet-20241022 vs. Gemini Experimental 1206 | -467 | **** | <0.0001 |
| claude-3-5-sonnet-20241022 vs. gpt-4o-2024-11-20 | -743.2 | **** | <0.0001 |
| claude-3-5-sonnet-20241022 vs. o1-preview-2024-09-12 | -639.4 | **** | <0.0001 |
| claude-3-5-sonnet-20241022 vs. Qwen-Max-2025-01-25 | -264.7 | **** | <0.0001 |
| deepseek-reasoner vs. Doubao-1.5-Pro-256k | 504.9 | **** | <0.0001 |
| deepseek-reasoner vs. Gemini Experimental 1206 | 192 | **** | <0.0001 |
| deepseek-reasoner vs. gpt-4o-2024-11-20 | -84.12 | ns | 0.4042 |
| deepseek-reasoner vs. o1-preview-2024-09-12 | 19.66 | ns | >0.9999 |
| deepseek-reasoner vs. Qwen-Max-2025-01-25 | 394.3 | **** | <0.0001 |
| Doubao-1.5-Pro-256k vs. Gemini Experimental 1206 | -312.9 | **** | <0.0001 |
| Doubao-1.5-Pro-256k vs. gpt-4o-2024-11-20 | -589 | **** | <0.0001 |
| Doubao-1.5-Pro-256k vs. o1-preview-2024-09-12 | -485.3 | **** | <0.0001 |
| Doubao-1.5-Pro-256k vs. Qwen-Max-2025-01-25 | -110.6 | * | 0.0439 |
| Gemini Experimental 1206 vs. gpt-4o-2024-11-20 | -276.1 | **** | <0.0001 |
| Gemini Experimental 1206 vs. o1-preview-2024-09-12 | -172.4 | **** | <0.0001 |
| Gemini Experimental 1206 vs. Qwen-Max-2025-01-25 | 202.3 | **** | <0.0001 |
| gpt-4o-2024-11-20 vs. o1-preview-2024-09-12 | 103.8 | ns | 0.0815 |
| gpt-4o-2024-11-20 vs. Qwen-Max-2025-01-25 | 478.5 | **** | <0.0001 |
| o1-preview-2024-09-12 vs. Qwen-Max-2025-01-25 | 374.7 | **** | <0.0001 |
